# Imaging of Mitochondrial and Non-Mitochondrial Responses in Cultured Rat Hippocampal Neurons Exposed to Micromolar Concentrations of TMRM

**DOI:** 10.1371/journal.pone.0058059

**Published:** 2013-03-04

**Authors:** Andrew Monteith, William Marszalec, Philip Chan, Juliette Logan, Weiming Yu, Nicholas Schwarz, David Wokosin, Philip Hockberger

**Affiliations:** 1 Department of Physiology, Feinberg School of Medicine, Northwestern University, Chicago, Illinois, United States of America; 2 Department of Molecular Pharmacology and Biological Chemistry, Feinberg School of Medicine, Northwestern University, Chicago, Illinois, United States of America; University G. D'Annunzio, Italy

## Abstract

Tetramethylrhodamine methyl ester (TMRM) is a fluorescent dye used to study mitochondrial function in living cells. Previously, we reported that TMRM effectively labeled mitochondria of neurons deep within mouse brain slices. Use of micromolar concentration of dye, which was required to get sufficient staining for two-photon imaging, resulted in typical fluctuations of TMRM. With prolonged exposure, we recorded additional responses in some neurons that included slow oscillations and propagating waves of fluorescence. (Note: We use the terms “fluctuation” to refer to a change in the fluorescent state of an individual mitochondrion, “oscillation” to refer to a localized change in fluorescence in the cytosol, and “wave” to refer to a change in cytosolic fluorescence that propagated within a cell. Use of these terms does not imply any underlying periodicity.) In this report we describe similar results using cultured rat hippocampal neurons. Prolonged exposure of cultures to 2.5 µM TMRM produced a spontaneous increase in fluorescence in some neurons, but not glial cells, after 45–60 minutes that was followed by slow oscillations, waves, and eventually apoptosis. Spontaneous increases in fluorescence were insensitive to high concentrations of FCCP (100 µM) and thapsigargin (10 µM) indicating that they originated, at least in part, from regions outside of mitochondria. The oscillations did not correlate with changes in intracellular Ca^2+^, but did correlate with differences in fluorescence lifetime of the dye. Fluorescence lifetime and one-photon ratiometric imaging of TMRM suggested that the spontaneous increase and subsequent oscillations were due to movement of dye between quenched (hydrophobic) and unquenched (hydrophilic) compartments. We propose that these movements may be correlates of intracellular events involved in early stages of apoptosis.

## Introduction

Tetramethylrhodamine methyl ester (TMRM) is a cationic, membrane permeable, fluorescent dye that stains mitochondria in many types of cultured cells [1]. While the mechanisms underlying cellular uptake and mitochondrial staining are not completely understood, there is persuasive evidence that the dye accumulates in mitochondrial membranes where the fluorescence is substantially quenched [2], [3]. Nevertheless, microscopic analyses revealed that mitochondria exposed to TMRM are fluorescent indicating that at least part of the dye remains unquenched [4]–[10].

Several groups have provided evidence that unquenched dye partitions within the mitochondrial intermembranous space driven by its Nernst potential across the inner mitochondrial membrane [4], [5]. Isolated mitochondria stained with TMRM, or related dyes tetramethylrhodamine ethyl ester (TMRE) and rhodamine 123, display spontaneous and evoked fluctuations in fluorescence that directly correlate with changes in membrane potential across the inner mitochondrial membrane [6]–[9]. Since similar results have been obtained from mitochondria in cultured cells [5], [9], [10], these dyes have been used extensively to evaluate the metabolic status of living cells.

While most studies to date have employed TMRM to study either isolated mitochondria or mitochondria in cultured cells, it would be useful to study mitochondrial function under more physiological conditions. Recently, we reported that bath applied TMRM readily stained mitochondria in neurons deep within rodent brain slices, and that stained mitochondria displayed fluctuations in fluorescence intensity similar to changes reported in cultured cells [11], [12]. Since brain slices are typically 200–300 µm in thickness and remain viable for only a few hours, we employed much higher concentrations of dye (10–25 µM) than is usually applied to cultured cells in order to accelerate the rate of staining and imaging of slices. While this approach provided an effective means of labeling mitochondria (yielding robust staining within 30–45 minutes), we discovered that some neurons displayed fluorescent responses that have not been reported previously. These responses and their characterizations are the focus of this paper.

In this study we used cultured neurons, instead of brain slices, for the following reasons: (1) cultured neurons displayed similar responses; (2) cultured neurons displayed the responses for several hours in physiological saline at room temperatures; (3) cultured neurons possessed planar geometries that facilitated whole cell imaging; and (4) cultured neurons were easier to load with Ca-sensitive dyes. Thus, cultured neurons provided several advantages without compromising the relevance to our brain slice data. A preliminary report of this work has been presented previously [13].

## Materials and Methods

### Ethics Statement

This study was carried out in strict accordance with the recommendations in the Guide for the Care and Use of Laboratory Animals of the National Institutes of Health. The protocol was approved by the Animal Care and Use Committee of Northwestern University (Approval Number: 2010-1151-1). Animal surgeries were performed under sodium pentobarbital anesthesia, and all efforts were made to minimize suffering.

### Primary Cultures

Rat hippocampal neurons were cultured using a modification of a technique described previously [14.15]. Hysterectomies were performed on pregnant Sprague-Dawley rats under sodium pentobarbital anesthesia on gestation day-18. Hippocampi were removed under a microscope, pooled, enzymatically digested with 0.25% trypsin (Sigma-Aldrich), mechanically triturated, and dissociated cells were suspended in Neurobasal medium with B-27 supplement (Gibco). One week earlier, 12 mm glass coverslips were treated with poly-L-lysine (Sigma-Aldrich) and grown to confluence with brain-derived glial cells. Dissociated hippocampal cells were plated onto glia-covered coverslips at a density of 100,000 cells/ml. After 3 hrs, coverslips were transferred to single wells of a 24-well plate containing fresh media. Cultures were maintained in a humidified atmosphere of 5% CO_2_ at 37°C for up to 4 weeks. All experiments were performed using cultures between 12 and 30 days *in vitro*. Previous reports have confirmed that neurons grown under these conditions developed morphological and electrophysiological properties that are consistent with mature neurons [15].

### Solutions and Dye Labeling

All experiments were performed at room temperature, unless specified otherwise. Cultures were bathed in physiological saline with the following composition (in mM): 125 NaCl, 2.5 KCl, 2 CaCl_2_, 1 MgCl_2_, 25 NaHCO_3_, 1.25 NaH_2_PO_4_, 25 glucose and pH adjusted to 7.4 with 95/5 mixture of O_2_/CO_2_. For 2P experiments, cultures were perfused with dyes and drugs using a peristaltic pump at 1 ml/min. For 1P experiments, dyes and drugs were added directly to cultures.

Stock solutions of TMRM and Flou-3 AM (10^−3^ M, Invitrogen) were prepared in 100% DMSO. Hoechst 33258 stock solution (200 mg/ml, Invitrogen) was prepared in 100% ethanol. All working solutions were prepared in physiological saline: 0.1–25 µM TMRM (Sigma-Aldrich, ≥95% purity), 1–100 µM FCCP (Sigma-Aldrich, ≥98% purity) and 10 µM thapsigargin (Sigma-Aldrich, ≥98% purity).

For Ca^2+^ experiments, cultured cells were incubated in 5 µM Fluo3 AM (Invitrogen) for 1 hr at 37°C, and then post-incubated in physiological saline for 1 hr at 37°C before imaging at room temperature.

### Mitochondrial Function Tests

Cultures were exposed to low and high concentrations of FCCP and/or thapsigargin to inhibit mitochondrial function. FCCP [carbonyl cyanide 4-(trifluoromethoxy) phenylhydrazone] is a H^+^ ionophore and uncoupler of oxidative phosphorylation capable of depolarizing plasma and mitochondrial membranes at nanomolar concentrations [16]. At higher concentrations, it can cause additional consequences including altering intracellular calcium and sodium dynamics [17], [18], decrease intracellular pH and inhibit inward rectifier K^+^ current [19] as well as influence neurotransmission, synaptic plasticity and neurodegeneration [20]. Consequently, our results obtained using micromolar concentrations of FCCP must be interpreted with caution as non-mitochondrial effects may have contributed to our observations.

Thapsigargin, a derivative of the plant *Thapsia garganica*, inhibits the Ca-ATPase (Ca pump) in sarcoplasmic/endoplasmic reticulum in cells at nanomolar concentrations [21]. Inhibition of the Ca-ATPase causes elevation of cytosolic Ca that can result in physiological consequences [22]. At higher concentrations, thapsigargin can depolarize mitochondrial membrane potential [23] and induce ER stress responses in cells [24]. Thus, as with FCCP, results with high concentrations of thapsigargin must be interpreted with caution.

### Live Cell Apoptosis Assay

Cultures were exposed to Millipore-filtered solutions (0.22 µm) containing TMRM and/or drugs for 1 hr at 37°C (except the experiment involving different durations of exposure to TMRM). After treatment, solutions were removed and growth media reapplied under sterile conditions, and cultures were post-incubated for 18 hours at 37°C (except for the experiment involving analysis at different time points after exposure). Cells were then stained with 2 mg/ml bisbenzimide (Hoechst 33258, Sigma-Aldrich) for 20 min at room temperature. Coverslips were subsequently washed in saline and imaged using 2P microscopy. Apoptotic cells were identified as brightly fluorescent nuclei under UV excitation indicating DNA fragmentation [25], [26]. Cell survivability was calculated as the percentage of live, unstained cells (± SD) in five microscopic fields per treatment.

### Two-photon (2P) Imaging

Two-photon microscopy is a non-linear optical technique employed to visualize fluorescent cells and tissues while minimizing phototoxicity of cells and photobleaching of dyes [27]. It accomplishes these advantages by limiting excitation of the sample to a discrete focal volume, typically 1–2 cubic microns, in the focal plane. For our purposes, it also enabled a direct comparison with our previous work in brain slices where 2P imaging was essential for imaging mitochondria in cells deep inside tissue [11], [12].

Coverslips with neurons were imaged in disposable 35 mm Petri dishes on the stage of an upright Olympus BX-51WIF microscope. The microscope was part of an Ultima two-photon (2P) imaging system (Prairie Technologies) possessing direct, dual PMT detection. All two-photon experiments used either an Olympus LUMPLFL 40×/0.8 NA or 60×/0.9 NA water-dipping objective. Image acquisition was controlled using *PrairieView* software that enabled single scans as well as time-lapse imaging. Image acquisition parameters were 4 µs/pixel dwell time, and 512×512 pixels^2^ per image. For time-lapse studies, images were acquired every 1–1.5 sec for up to 70 min. Faster scanning (0.125 sec/image) was facilitated in some experiments by reducing the size of the image field.

The Ultima system was equipped with a Ti:sapphire pulsed infrared laser (Mira 900, Coherent) pumped by a 532 nm solid state laser (5 W Verdi, Coherent). The Ti:sapphire laser generated excitation pulses at 76 MHz using a Kerr lens mode-locking system that enabled an average pulse width of approximately 200 fsec between 800–835 nm. Laser power at the sample was controlled using a Pockels’ cell modulator (Conoptics model M350-50-02 BK) yielding average powers in the range of 0.2 to 40 mW at the sample plane. Laser power was monitored along the beam path by a silicon photodiode (Hamamatsu SK 3348-44), and average power at the sample plane was calibrated using a thermopile detector (Molecular Devices) mounted beneath the objective lens. All experiments were performed using beam intensities less than 3 mW at the sample in order to avoid the non-linear, laser-induced phototoxicity regime [28].

The two-photon excitation peak of TMRM is 830 nm, [29] similar to the 2P cross section for rhodamine B [30]. We used 820 nm (6 nm bandwidth) laser output to excite TMRM in our experiments. While not optimal excitation for TMRM, this wavelength minimized potential blue-light toxicity [31]. The choice of 820 nm also optimized excitation of Fluo-3 imaging without compromising TMRM visualization. Simultaneous dual emission signals were passed through a chromatic reflector, 560 DCLPXR (Chroma Technologies), and filtered through either a 525/50 nm or 610/60 nm bandpass filter (Chroma Technologies).

### One-photon (1P) Ratiometric Imaging

Ratiometric imaging of TMRM stained cells was performed as described by Scaduto and Grotyohann [2]. This technique was developed to overcome the problem of binding of TMRM to mitochondrial membranes that complicates its use for measuring changes in mitochondrial membrane potential. Ratiometric measurements adjust for movements of dye between bound and unbound compartments thereby enabling quantitative measurements of mitochondrial membrane potential (also see [3]).

We used a custom-built ratiometric excitation imaging system that employed a fast scanning monochromator and high sensitivity camera detection system attached to an inverted Nikon TE300 microscope with CFi-60 PlanApochromat 60×/1.2 NA water-immersion objective. Cells were excited sequentially at 546 and 573 nm (5 nm bandwidth) at 100 msec intervals, and a ratio image was formed every 200 msec from the corresponding emission images. Regions of interest in the ratio images were plotted over time using NIH Image software.

The excitation source was a Cairn Optoscan UV-VIS monochromator (300 nm to 600 nm range with first order only, and bandwidth selectable from 1–30 nm) controlled with *WinFluor* software, provided by John Dempster (Strathclyde University, Scotland). The monochromator was attached to the epi-illumination port of the microscope. Filter cubes were acquired from Chroma Technologies to permit ratiometric imaging of different excitation wavelengths from the monochromator with a defined emission detection window. The TMRM filter cube had no excitation bandpass filter, a 585 DCLP separation chromatic reflector, and a 620/60 nm emission bandpass filter. The side port of the microscope (3× magnification) was fit with a Hamamatsu *ImagEM* (C3900) camera and used without multiplication gain. Camera pixel size was 7 µm, so bin 2 imaging was employed.

### Fluorescence Lifetime Imaging

Fluorescence lifetime imaging microscopy (FLIM) is a technique that measures a fluorophore’s decay rate, rather than its intensity, thereby eliminating differences in fluorescence due to variations of dye concentration within cellular compartments. In addition, it is useful for detecting fluorophores in different cellular compartments where decay rates vary due to differences in the local environment [32]. We combined FLIM with 2P imaging to take advantage of the high spatial resolution of 2P and the intensity-independent properties of FLIM.

Fluorescence lifetime images were acquired using a digital frequency domain FLIM module [33] integrated with the 2P imaging system. The output of one of the PMTs of the 2P imaging system (Prairie Technologies) was connected to the FLIM module (FastFLIM box, model A320, ISS Inc.) to enable data acquisition. This arrangement allowed us to conveniently screen for and record TMRM oscillations using the Prairie 2P system before and after FLIM measurements. The oscillation frequency of the Ti-sapphire laser (76 MHz) was used as the external clock for the FastFLIM box.

FLIM imaging utilized 810 nm excitation for two-photon excitation (also for screening and recording of TMRM oscillations), 40×/0.8 NA water dipping objective (Olympus), 10 msec pixel dwell time and 512×512 pixels^2^ per image (ZOOM). The phase stability of instrument was monitored using Coumarin 2.5 dye (Invitrogen) dissolved in ethanol. No significant phase drift was detected during the course of the experiments.

Intensity, phase and modulation images were collected using *VistaVision Suite* software (ISS Inc.), and fluorescence lifetimes were analyzed on-line using the phasor plot [34]. Single and average scan images were collected, and phase and modulation lifetimes were determined for individual cell bodies, cytoplasm, nuclei and mitochondria. The average median phase value was calculated during high and low TMRM fluorescent states.

### Image Processing

Image analyses were performed off-line using *NIH ImageJ*, *Microsoft Excel*, *MatLab* (The MathWorks, Natick, MA), and *Adobe Illustrator* software programs. Time-lapse movies were created using ImageJ and saved as high resolution.avi files. Compression of movies into.mp4 files at reduced resolution for publication required Apple QuickTime Player. Regions of interest (ROI) were defined and analyzed using ImageJ and copied to Excel for statistical analysis and graphing. *MatLab* was used for drawing curves and for calculating the Fast Fourier Transform (FFT) of oscillations. *Illustrator* was used to create the final figures.

2P data is presented as normalized intensity (each pixel value divided by the maximum pixel value) to facilitate simultaneous display of data in different cellular compartments. 1P intensity data is presented in arbitrary units divided by the maximum value per trace. Intracellular Ca^2+^ changes are presented as “fold” changes relative to baseline.

## Results

### Effects of Low Concentrations of TMRM

Exposure of hippocampal cultures to low concentrations of TMRM (50–500 nM) for 1–3 hours resulted in selective staining of mitochondria in both neurons and the underlying glial cells. With 2P time-lapse imaging, some mitochondria displayed spontaneous fluctuations in fluorescence, similar to what has been reported for cultured neuronal [9] and non-neuronal cells [5], [10]. These fluctuations persisted for up to an hour but could be rapidly eliminated by bath application of 1 µM FCCP (n = 5 coverlips).

**Figure 1:**
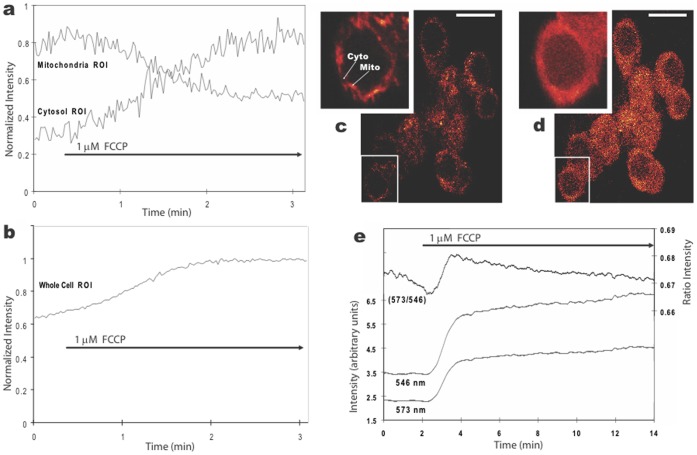
Changes in fluorescence in cultured hippocampal neurons stained with 100 nM TMRM and exposed to bath-applied 1 µM FCCP. **a, b.** Plots of average 2P fluorescence (normalized) versus time for five mitochondria and adjacent cytosolic regions (a), and eight whole cell bodies (b). The FCCP-induced loss of fluorescence in mitochondria correlated with simultaneous increases in fluorescence in adjacent cytosolic regions, whereas the whole cell fluorescence displayed an overall increase. **c, d.** 2P fluorescent images of the TMRM labeled cells before (c) and after two minutes in FCCP (d), with insets at higher magnification showing the diffuse distribution of dye after FCCP. Calibration bar is 20 µm. **e.** Plot of average 1P fluorescence, using excitation at two wavelengths (546 and 573 nm) and their ratio (573/546), for five cell bodies before and during FCCP exposure.

By adjusting the 2P excitation intensity appropriately and selecting a plane of focus that prevented excitation of the underlying glial cells, it was possible to measure FCCP-induced loss of fluorescence from individual mitochondrion and a corresponding increase in fluorescence in the adjacent cytosol (Fig. 1a). This result is similar to data reported for cultured Purkinje neurons [35] and supports the interpretation that uncoupling of mitochondrial membrane potential by FCCP facilitated release of dye from mitochondrial compartments. The overall increase in cellular fluorescence (Fig. 1b) was therefore most likely due to release of dye from the mitochondrial inter-membranous space (unquenched dye) and, possibly, release of quenched dye as it moved from within mitochondrial membranes to the cytoplasm.

We investigated the possibility that quenched dye contributed to the cytoplasmic fluorescence using 1P ratiometric imaging. This technique has been shown to detect unquenching of TMRM in isolated mitochondria [2] while at the same time normalizing responses to changes due to differences in dye concentration [36]. Exposure of cultures to 1 µM FCCP resulted in an increase in the 573/546 ratio in 5 of 5 cells examined (Fig. 1e), indicating that unquenching of TMRM occurred as it moved from mitochondrial membranes (quenched) to cytosolic (unquenched) compartments. Thus, these results support the idea that FCCP induced release of dye from both quenched and unquenched compartments. We rely on this dual interpretation to provide context and contrast with the results described in the following sections using higher concentrations of TMRM.

### Effects of High Concentrations of TMRM

**Figure 2:**
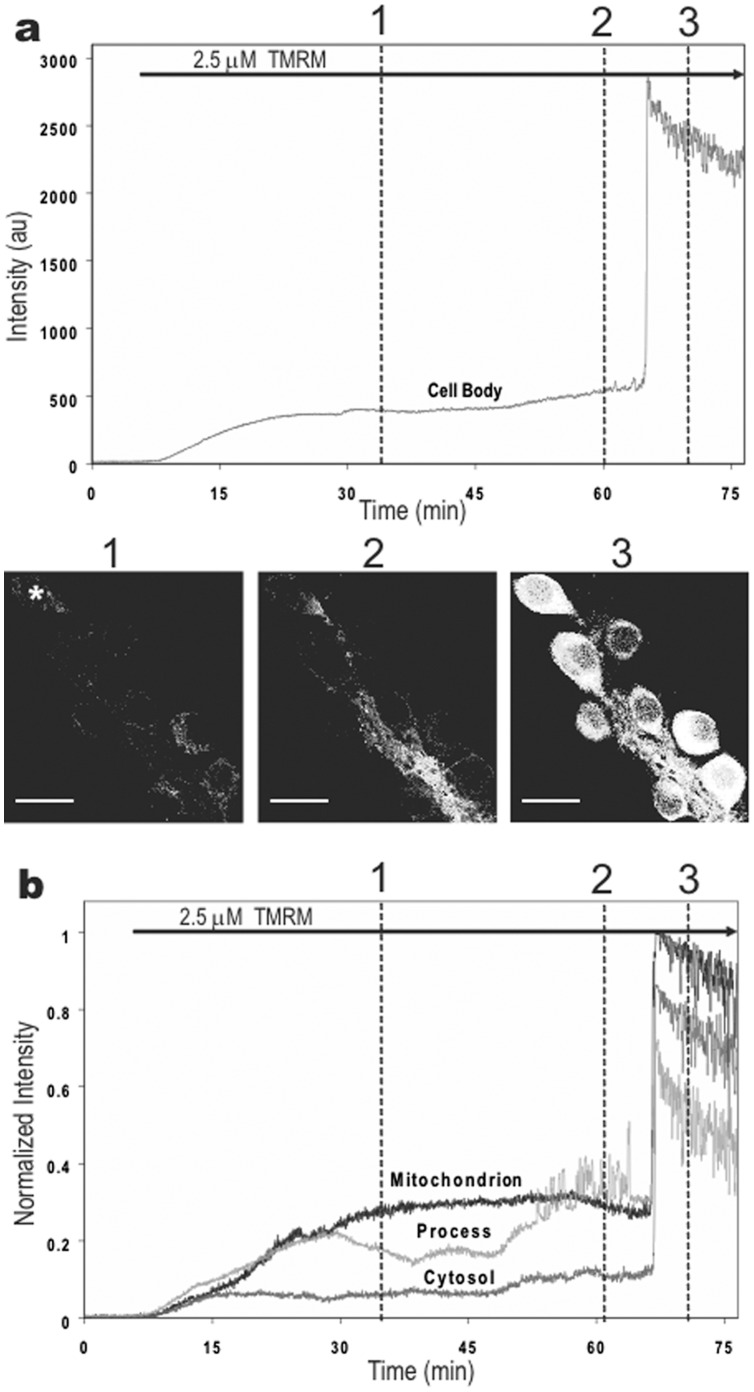
Time-course of 2P fluorescence in cultured hippocampal neurons exposed to 2.5 µM TMRM. Dye was applied at the start of recordings. The time-lapse movie of these results can be viewed in the supplemental figures (Movie S1). **a.** Plot and images of 2P fluorescence in a representative cell body (asterisk) at three time points marked by dotted lines in the graph. Notice the large increase in fluorescence just after the 1-hr mark. Calibration bar is 20 µm. **b.** 2P fluorescence (normalized) plotted for a mitochondrion, proximal neurite, and cytoplasm in the same cell. The delayed increase in fluorescence occurred simultaneously in all three compartments.

Exposure of hippocampal cultures to high concentrations of TMRM (1–25 µM) stained mitochondria selectively and quickly, reaching a plateau after 5–10 min (Fig. 2a). Spontaneous fluctuations in mitochondria fluorescence were similar to results obtained with low dye concentrations. By using low laser power to minimize background fluorescence (as the dye was present throughout) and to minimize photodynamic damage, we monitored mitochondrial fluctuations for 30–45 min in cells with no apparent toxicity.

After approximately 45–60 min, though, cellular fluorescence abruptly increased in some neurons (Fig. 2a, time point 3). This large spontaneous increase in fluorescence occurred simultaneously in all compartments of a cell (Fig. 2b), although there was no apparent coordination between cells (Movie S1).

The cause of this large spontaneous increase in cellular fluorescence was not immediately obvious. If it was due to TMRM release from mitochondria, then it would indicate that mitochondria have an immense capacity for storing TMRM as the emission signal often saturated our detectors. On the other hand, the spontaneous increase was usually preceded by a gradual increase in fluorescence in neurites where the number and density of labeled mitochondria appeared fewer than in cell bodies (Movie S1). While the initial increase in neurites may have resulted from their greater surface-to-volume ratio, we tested whether mitochondria contributed to this response by analyzing individual mitochondrion.

**Figure 3:**
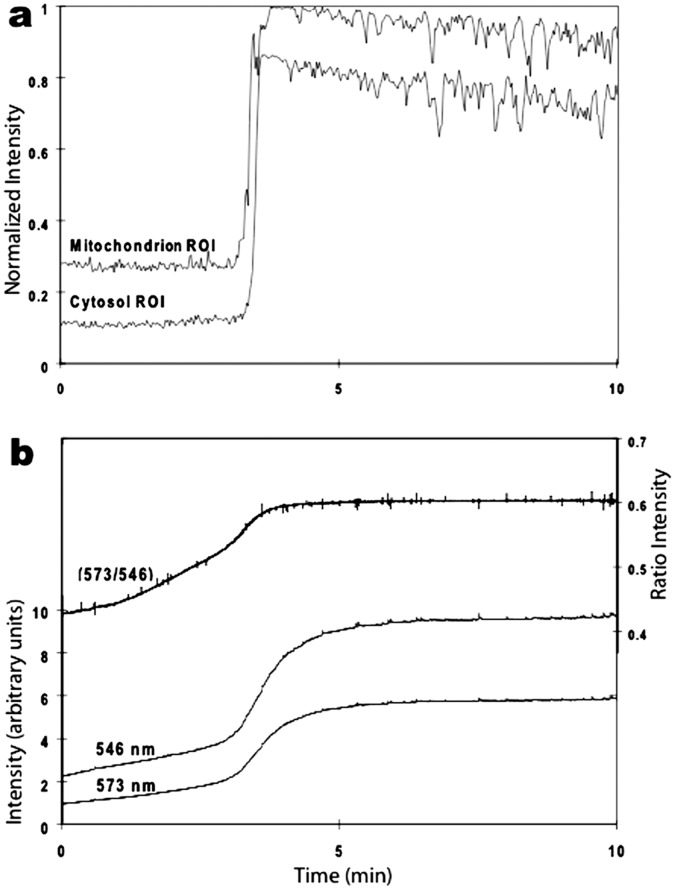
Detection of a large spontaneous increase in fluorescence in cultured hippocampal neurons after 1-hr exposure to 2.5 µM TMRM. **a.** Plot of 2P fluorescence (normalized) versus time for regions of interest (ROI) over a single mitochondrion and adjacent cytosolic region. The spontaneous increase in both compartments was markedly faster (and brighter) than the average FCCP-induced responses in Figure 1a. **b.** Plot of 1P fluorescence using excitation at two wavelengths (546 and 573 nm) and their ratio (573/546) before and during a spontaneous increase in TMRM fluorescence. The increase in whole cell fluorescence was slower than the individual mitochondrial response but similar to the average cellular response to FCCP shown in Figure 1b.

We recorded a simultaneous increase in *both* mitochondrial and adjacent cytosolic fluorescence during the spontaneous increase in cellular fluorescence (Fig. 3a). This result supported the idea that there was much greater release of TMRM by mitochondria than was found using low TMRM concentrations. The possibility that unquenching of TMRM contributed to this response was tested using 1P ratiometric imaging. The 573/546 ratio increased during the spontaneous increase in cellular fluorescence (Fig. 3b), thus, supporting the hypothesis that the large spontaneous increase in cellular fluorescence resulted from TMRM released from both quenched and unquenched compartments of mitochondria. The prospect that additional cellular compartments contributed to this signal is explored in the following sections.

Before proceeding, we would like to emphasize that the large spontaneous increase in cellular fluorescence did not occur in all cells. It was not seen, for example, in the bed of glial cells lying beneath neurons, nor was it present in some neurons even after several hours of very high concentrations of TMRM (25 µM). In glia and unresponsive neurons, TMRM remained restricted to mitochondria, which displayed their characteristic fluctuations throughout the recording period.

It is also worth mentioning that the large spontaneous increase in cellular fluorescence did not result from illumination of cells, which has been shown in other cell types [37], [38], because it was immediately present in neurons pre-incubated in dye for 1–2 hrs. In other words, the spontaneous elevation occurred regardless of whether we imaged the cells or not. There were variables that seemed to enhance the likelihood of obtaining the spontaneous increase in fluorescence (higher dye concentration, longer dye incubation period, older age of cultures, higher oxygen levels of the saline), but we did not test these systematically. Instead, we standardized these variables to ensure that they remained controlled during our experiments.

### High Concentrations of TMRM Induced Neuronal Apoptosis

**Figure 4:**
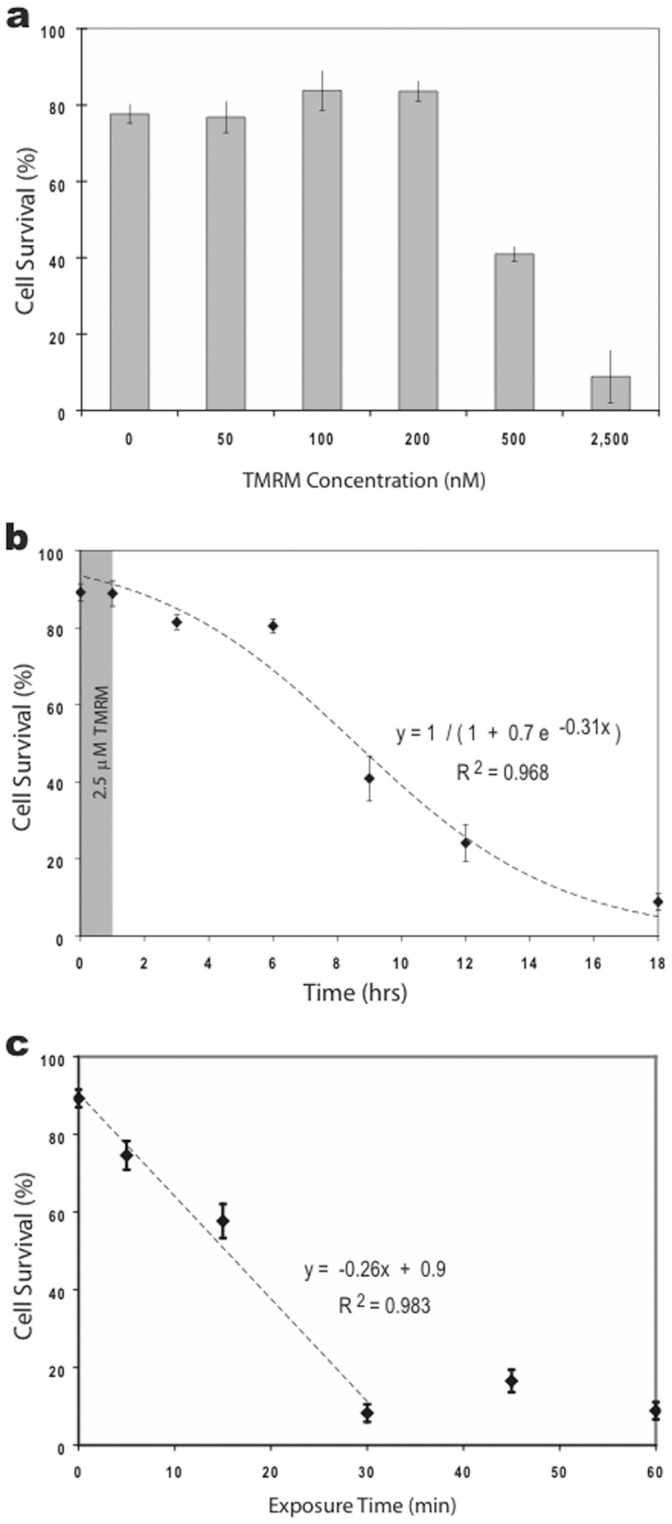
TMRM induced apoptosis in cultured hippocampal neurons. **a.** Histogram comparing the effects of different concentrations of TMRM concentrations on cell survivability. Survivability was approximately 80% in control solutions (saline or 2.5 µM DMSO) indicating that exposure to saline, but not DMSO, for 1 hr had a small but measurable effect. Nevertheless, exposure to low concentrations of TMRM (50–200 nM) for 1 hr were not significantly different from controls. Higher concentrations (0.5 & 2.5 µM) induced substantially greater cell death. **b.** Graph of cell survivability over time following a 1-hr exposure to 2.5 µM TMRM (shaded area). The slow response time is indicative of apoptosis and ruled out necrosis. **c.** Graph of cell survivability 18 hrs after exposure to 2.5 µM TMRM for different durations (5, 15, 30, 45 and 60 min).

One obvious interpretation of the results in the previous section is that the spontaneous increase in fluorescence was a toxic response due to prolonged exposure of TMRM at high concentrations. To investigate this possibility, we tested cultures exposed to different concentrations of dye for one hour followed by staining for apoptosis with bisbenzimide (Hoechst 33258) 18 hrs later. As shown in figures 4a, low concentrations of TMRM (50–200 nM) did not induce apoptosis, whereas higher concentrations (0.5 & 2.5 µM) enhanced apoptosis (K_D_ = 500 nM). The slow time course of the response was also consistent with an apoptotic mechanism (Fig. 4b), as was the observation that shorter exposure times were less effective. Nevertheless, as little as 5 min of 2.5 µM TMRM was sufficient to induce apoptosis in 15% of the neuronal population (Fig. 4c).

**Figure 5:**
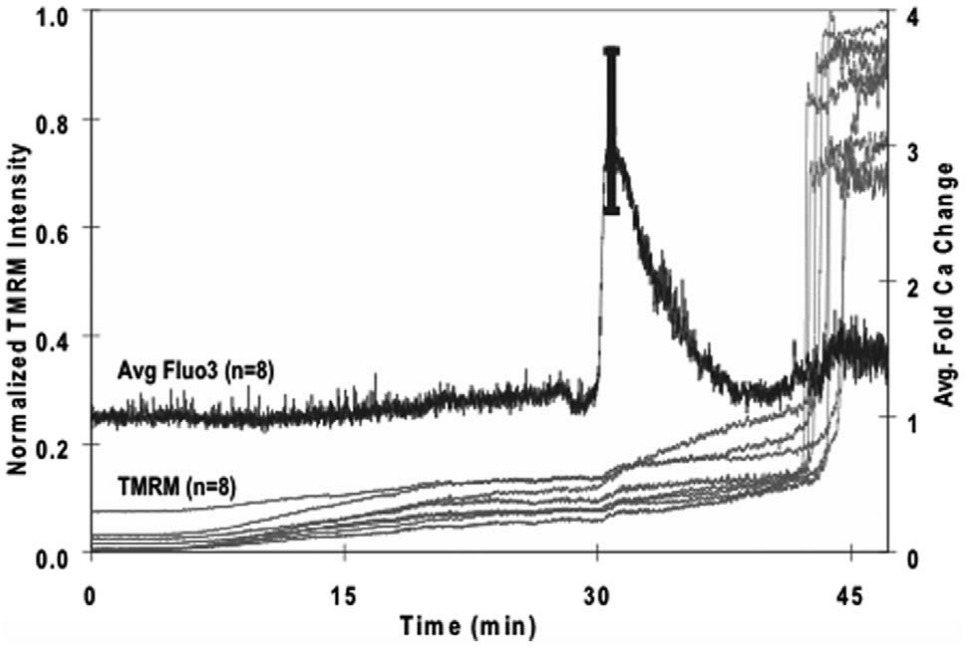
Simultaneous dual-probe fluorescence measurements of cultured hippocampal neurons pre-labeled with Flou-3 AM and exposed to 2.5 µM TMRM. Plot of average 2P fluorescence for both dyes versus time. TMRM plot shows individual traces for eight neurons, and Flou-3 plot shows the average normalized response for the same eight neurons. The Flou-3 signal was averaged because the Ca responses were synchronized in all eight neurons, unlike the TMRM responses. A large spontaneous increase in intracellular Ca occurred approximately 15 min prior to the large spontaneous increase in TMRM fluorescence.

For reasons that will become apparent below, we measured the Ca^2+^ levels in cells exposed to TMRM and found additional evidence for apoptosis. Neurons were pre-labeled with the Ca-sensitive dye, Fluo-3 AM, then exposed to 2.5 µM TMRM for one hour. Approximately 30 minutes after the start of TMRM, there was a spontaneous, synchronized elevation of intracellular Ca^2+^ level in those neurons that subsequently displayed a large, spontaneous increase in TMRM fluorescence (Fig. 5). That is, there was a one-to-one correspondence between cells that displayed both responses. The Ca^2+^ response surprised us because it was obtained *before* the bisbenzimide experiments. It was only later that we recognized that this Ca^2+^ response was correlated with apoptosis.

### High Concentrations of TMRM Induced Fluorescent Oscillations and Waves

Over time, the large spontaneous increase in TMRM fluorescence dissipated in some neurons. Time-lapse 2P imaging revealed two types of subsequent changes in fluorescence: local oscillations and propagating waves. The former were changes in fluorescence within distinct regions of a cell (e.g., cell body, neurite), whereas the latter were transverse waves that propagated within cells (e.g., from neurite 1 to cell body to neurite 2). Unlike previous reports of illumination-induced oscillations that persisted for a few minutes followed by irreversible loss of fluorescence [37], [38], the oscillations and waves we measured persisted for several hours.

**Figure 6:**
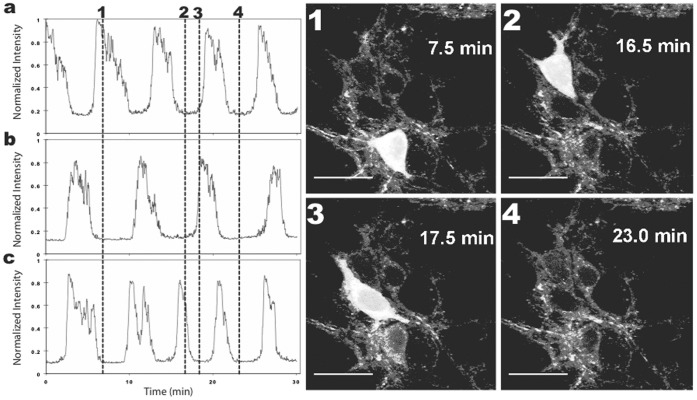
Oscillations in TMRM fluorescence occurred in cultured hippocampal neurons. Cells were stained with 25 µM TMRM for 15 min and examined 3 hrs later. Staining, washing and imaging were all performed at room temperatures. Graphs of fluorescence versus time on the left are for the three cells imaged on the right. Graph a is for the cell in image 1, graph b for the cell in image 2, and graph c for the cell in image 3. Dotted lines in the graph show the approximate time points when the images were taken. Calibration bar is 20 µm. The time-lapse movie of these results is shown in Movie S2.

Local TMRM oscillations occurred in both regular and irregular patterns. An example of a regular pattern is shown in Fig. 6 for three neurons located within a cluster of neurons. The oscillations were slow and unsynchronized between cells. Time-lapse recording revealed that local oscillations correlated with fluorescent waves that propagated within neurons in random directions (Movie S2). The waves originated in unpredictable locations, sometimes within cell bodies and sometimes within neurites. Such behavior gave the appearance of a diffusive process, although the waves were much slower than would be expected from simple diffusion of dye between compartments (see Discussion). The average wave velocity for the three cells in Fig. 6 was 0.7±0.2 µm/sec at room temperature (20–22°C).

The majority of oscillating neurons displayed irregular patterns that sometimes switched from fluorescent to non-fluorescent states for extended periods of time (Figure S1). When whole cell fluorescence decreased, some mitochondria remained fluorescent and continued to display fluctuations. In a subset of these cells, clusters of mitochondria sometimes generated oscillations that initiated propagating waves of fluorescence. These results suggested that some mitochondria were still functioning and could contribute to the generation of oscillations and waves.

Oscillations and waves were detected in both the cytoplasm and nuclei of neurons. The latter were evaluated by optically sectioning cell bodies to determine the size of the nucleus. By systematically varying the size of steps in the Z-axis, we determined that the 2P excitation focal depth was approximately 2 µm (using the 40× objective) and that most neuronal nuclei were 10–12 µm in diameter. By choosing the plane of focus appropriately, we were able to confirm that the waves and oscillations permeated nuclei. On the other hand, we saw no evidence of fluorescence changes originating within nuclei or across the plasma membrane. All of our results are consistent with an intracellular origin of the fluorescent phenomena.

We assessed whether unquenching of TMRM contributed to oscillations using 1P ratiometric imaging. The 573/546 ratio increased and decreased during oscillations, identical to the results obtained during the initial spontaneous increase in cellular fluorescence (cf. Fig. 3b), indicating that unquenching of dye correlated with the oscillations. This suggested that the dye was moving between quenched and unquenched compartments during the different phases of oscillation. We will provide additional support for this hypothesis using FLIM (see below).

### TMRM Oscillations Persisted in FCCP and Thapsigargin

**Figure 7:**
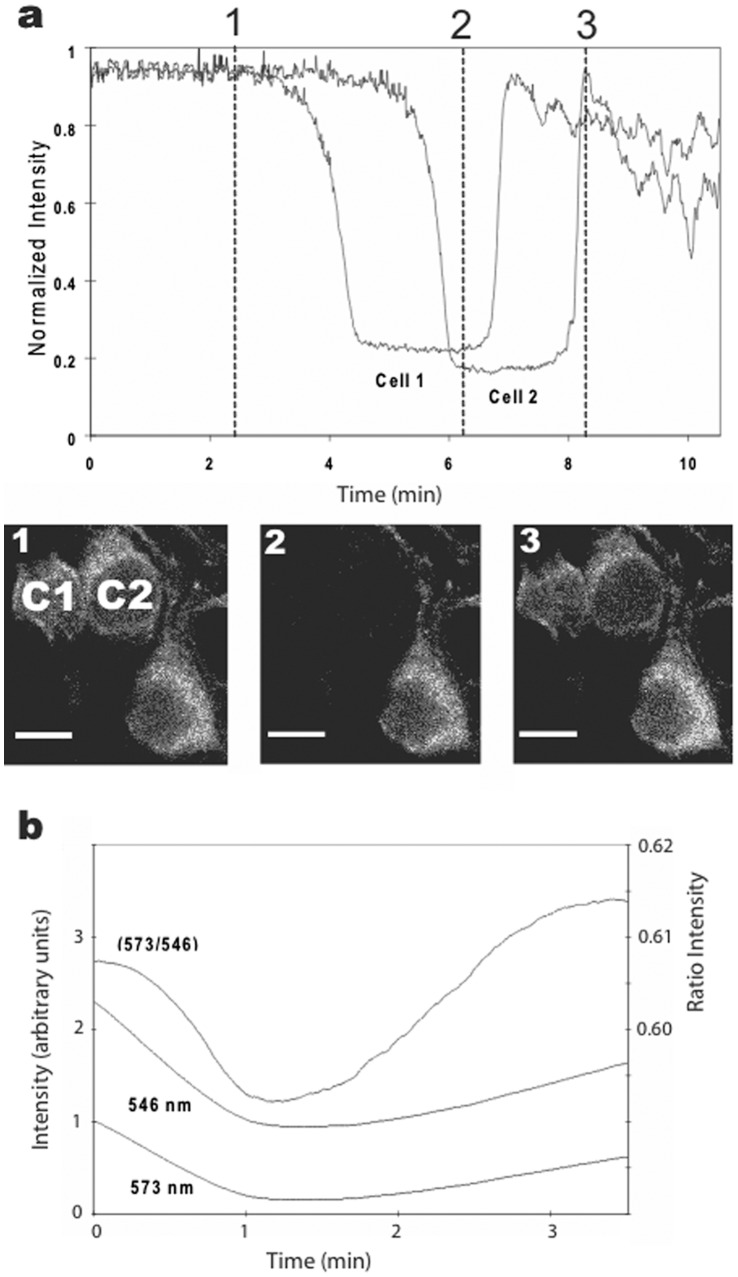
Oscillations in TMRM fluorescence were not dependent upon functioning mitochondria. Cultures were pre-treated with 100 µM FCCP for 15 min and then imaged after one hour in 2.5 µM TMRM. **a.** Plots of 2P fluorescence (normalized) versus time for two neurons (c1 and c2) displayed oscillations similar to those in neurons without FCCP exposure. Calibration bar is 20 µm. **b.** Plot of 1P fluorescence (normalized) versus time for an oscillating neuron. The ratio changes are consistent with dye movement between hydrophobic and hydrophilic compartments (see text for details).

We investigated the possibility that cyclical uptake and release of TMRM by mitochondria generated the oscillations. Neurons were pre-incubated in FCCP (1**,** 10, or 100 µM), thapsigargin (10 µM), or both for 15 min. The high concentration of each drug ensured maximal inhibition of mitochondrial function [23], [39]. As expected, mitochondria did not label with 2.5 µM TMRM under these conditions even after one hour of exposure. To our surprise, however, the large spontaneous increase in cellular fluorescence still occurred in many neurons, and some displayed oscillations (Fig. 7a). Occasionally, we saw propagating waves, but we have insufficient data to assess whether FCCP altered their appearance or frequency because waves were not as common as oscillations. Nevertheless, this data provides compelling evidence that functional mitochondria were *not* required for the spontaneous increases in cellular fluorescence or oscillations, although they did contribute (as demonstrated above).

Next, we assessed whether pre-treatment with FCCP altered the unquenching response using 1P ratiometric imaging. We tested both the large spontaneous increase in cellular fluorescence as well as oscillations. Pre-treatment with FCCP had no noticeable effect on either response. The ratiometric data for the spontaneous increase in cellular fluorescence looked identical to data recorded in the absence of FCCP, and the ratio data for oscillations followed the single wavelength data faithfully (Fig. 7b). These results support the argument that unquenching of dye from non-mitochondrial compartments contributed to the spontaneous increase in fluorescence as well as to subsequent oscillations.

Since TMRM was present in the bath throughout these experiments, it is possible that enhanced uptake of the dye into cells contributed to the spontaneous increase in fluorescence. This seems unlikely, though, based upon the 1P ratiometric results. A more plausible explanation is that TMRM was released from intracellular compartments where the dye was quenched, and that the increase was associated with a coordinated, spontaneous release of dye from these compartments (mitochondrial and non-mitochondrial). While the identity and location of the non-mitochondrial compartments remain a mystery, TMRM waves readily permeated the nucleus indicating that dye readily passes through nuclear membranes (pores).

### TMRM Oscillations did not Correlate with Changes in Intracellular Ca^2+^

**Figure 8:**
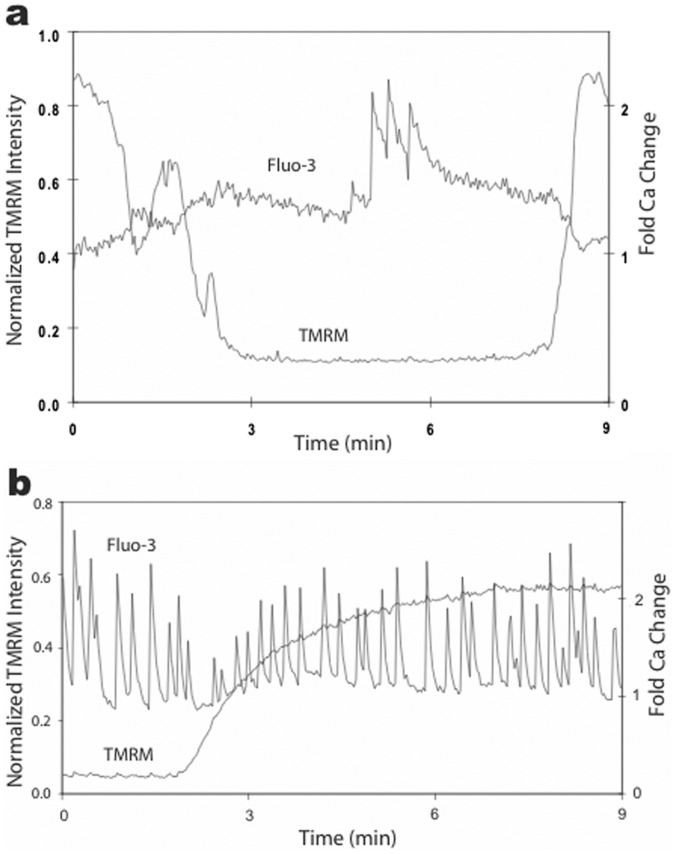
Oscillations in TMRM fluorescence did not correlate with changes in intracellular Ca^2+^. Simultaneous measurements of Fluo-3 and TMRM in two different neurons (a, b) revealed no correlation between these signals (Movie S3). **a.** This neuron generated several Ca^2+^ spikes during the low fluorescent state of a TMRM oscillation. **b.** Another neuron displayed sustained Ca^2+^ spiking that was unperturbed by bath application of TMRM. Similar results were obtained in all cells examined (n = 8).

TMRM oscillations have been associated with oscillations in intracellular Ca^2+^ in other cell types [40], [41]. Therefore, we tested this possibility using hippocampal neurons pre-loaded with the Ca-sensitive dye Fluo-3 (n = 8). We made three types of observations that revealed no correlation between TMRM oscillations and intracellular Ca^2+^ changes (Fig. 8): Ca^2+^ changes occurred in the absence of TMRM oscillations; TMRM oscillations occurred in the absence of Ca^2+^ changes; there was no correspondence between Ca^2+^ changes and TMRM oscillations when both were present. In addition, the Ca^2+^ responses were much faster and synchronized between adjacent cells, whereas TMRM responses were slow and asynchronized. In short, we found no evidence that TMRM oscillations in hippocampal neurons were correlated with changes in intracellular Ca^2+^. These results also indicated that hippocampal neurons retained their electrical excitability in the presence of TMRM, and that TMRM oscillations were not correlated with membrane excitability.

We tried two additional approaches to assess the role of Ca^2+^ in TMRM oscillations, but both approaches inhibited oscillations. First, we lowered external Ca from 2 mM to 0.1 mM (and raised external Mg from 1 mM to 3 mM to maintain osmotic equilibrium, and pretreated cultures with 10 µM FCCP to inhibit mitochondria) using push-pull perfusion. TMRM oscillations were inhibited within 30 sec of exposure, and they returned after 1–2 min back in physiological saline (n = 3). Second, a patch pipet containing 10 mM EGTA (plus 100 nM TMRM and standard intracellular saline) was sealed onto a neuron displaying TMRM oscillations (also see [12]). Upon breaking into the cell and recoding an intracellular membrane potential, oscillations immediately ceased and fluorescence remained stable thereafter, i.e., for the duration of the recording (typically 15 min). Since both approaches inhibited TMRM oscillations, neither allowed further evaluation of the role of Ca^2+^ in the oscillations.

Of course these results do not rule out the possibility that Ca^2+^ needs to be present at physiological levels for oscillations to occur. Nevertheless, even though Ca^2+^ may need to be present, our live imaging experiments demonstrate Ca^2+^ levels in the cell do not correspond with TMRM oscillations. Other cellular processes dependent on physiological levels of Ca^2+^ may be driving these TMRM oscillations, but since both approaches inhibited TMRM oscillations, neither allowed further evaluation of the role of Ca^2+^ in the oscillations.

### TMRM Oscillations Correlated with Different Fluorescent Lifetimes

**Figure 9:**
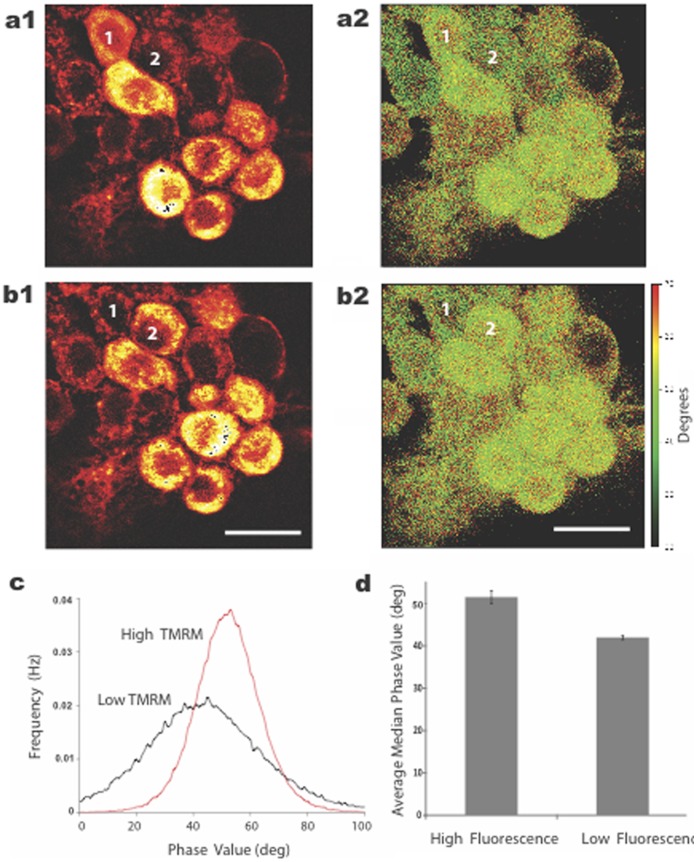
Oscillations in TMRM fluorescence correlated with changes in fluorescence lifetime imaging of the dye. **a, b.** FLIM intensity images (a1, b1) and the corresponding phase modulation images (a2, b2) of a cluster of neurons are shown for different oscillatory states (high and low florescence) 43 min apart. Two cells (numbered in white) were in opposite states at the two time points and provided a one-for-one correlation between lifetime and oscillatory state. The time-lapse movie of these results, captured between a and b, is shown in Figure S1. **c.** Cells in the low fluorescent state (black trace, average of 5 cells) displayed a phase shift of fluorescence relative to cells in the high fluorescent state (red trace, average of 5 cells) indicating different local environments of the dye. **d.** The average median intensity value of the phase data was also significantly different between cells in low and high fluorescent states. Calibration bar is 20 µm.

Since 1P ratiometric measurements supported the hypothesis that TMRM was moving between quenched (hydrophobic) and unquenched (hydrophilic) compartments during oscillations, we tested whether there was a corresponding change in fluorescence lifetime of the dye. Such a correlation would also be consistent with dye movement between these compartments. As shown in figure 9, indeed, there was a significant difference in the average lifetime of the dye during the phases of TMRM oscillations. Due to the complex properties of TMRM fluorescence (i.e., multi-exponential relaxation rates), we were unable to discern the actual component lifetimes using one modulation frequency (76 MHz). Nevertheless, neurons in the low fluorescent state displayed a significant phase difference (Fig. 9c), as well as a change of modulation value (data not shown) of the dye relative to neurons in the high fluorescent state. This result supports the idea that TMRM oscillations resulted from dye movement between hydrophobic and hydrophilic compartments.

In between FLIM measurements, we performed time-lapse 2P imaging and confirmed that the cells were indeed oscillating (Movie S3). The movie also demonstrates the diversity of cellular responses we observed, as some neurons displayed elevated fluorescence without oscillations while others displayed only mitochondrial fluctuations. This diversity is representative of what we found in the course of examining the effects of high concentrations of TMRM in cultured hippocampal neurons.

## Discussion

This report documents several new findings related to the use of TMRM for studying mitochondrial function in cultured neurons as well as in brain slices. First, we found that most cultured hippocampal neurons tolerated 2.5 µΜ concentration of dye for up to 15 min without inducing apoptosis, a fairly sensitive toxicity assay (since it takes many hours to be revealed). Some neurons continued to display mitochondrial fluctuations even after several hours of micromolar dye concentration, consistent with the relatively low toxicity of TMRM compared to other mitochondrial dyes [2].

Hippocampal neurons were not, however, immune to the dye’s toxic effects. As shown in Figure 4c, some neurons were affected by fairly short exposures. Nevertheless, the majority of neurons were resistant to apoptosis when exposed for 15 min or less. Extrapolating to brain slices, it seems reasonable to propose the use of micromolar concentrations of TMRM for studying mitochondrial function in brain slices as long as the exposure is kept reasonably short (i.e., no more than 15 min after the start of labeling) and so long as fluorescence remains restricted to mitochondria.

The main focus of this paper, however, described and analyzed the source of the spontaneous increase in fluorescence of hippocampal neurons that occurred after prolonged exposure to high concentrations of TMRM. Our pharmacological data indicated that the increase resulted from spontaneous release of dye from both mitochondrial and non-mitochondrial compartments. Ratiometric and FLIM data provided direct support for the hypothesis that the subsequent oscillations were caused by cyclical movement of dye between quenched (hydrophobic) and unquenched (hydrophilic) compartments. Thus, while mitochondria were involved, they were not the only compartments contributing to the oscillatory responses.

While the identity of the non-mitochondrial compartment(s) is unknown, it is clear that the dye was completely quenched within that compartment (i.e., it was not detectable with our imaging methods). Our conjecture that high concentrations of TMRM caused dye to bind to non-mitochondrial compartments is not surprising since Scaduto and Groyohann [2] noted that TMRM is amphipathic and readily partitions in cellular membranes. What is surprising, though, is that time-lapse imaging of fluorescent waves suggested that nuclei possessed a quenched pool of dye (Fig. 6). Thus, both nuclear and cytoplasmic compartments may possess quenched dye, an observation that may aid future studies aimed at identifying the source of the spontaneous increase in fluorescence.

In our experiments we took advantage of ratiometric imaging to eliminate several technical limitations of previous single wavelength measurements, e.g., photobleaching of dye, drifting in the field of view, fluctuations in illumination intensity, and differences in concentration of dye among cells and compartments [36]. It also took advantage of shifts in dye spectra, when present, to amplify fluorescent responses [3]. Scaduto & Grotyohann [2] used the spectral shift of TMRM to amplify responses of mitochondria to respiratory uncoupling agents (CCCP, DNP). These agents induced an increase in fluorescence with 546 nm excitation, and a small decrease in fluorescence with 573 nm excitation, resulting in a decrease in the 573/546 emission ratio. These experiments were performed using acutely isolated rat heart mitochondria and measured using a fluorometer.

Our ratiometric measurements were performed using a microscope-based imaging system that tracked TMRM stained mitochondria in living cells. This is noteworthy as our ratiometric results differed in one important way from Scaduto & Grotyohann’s. We found that FCCP induced an increase in fluorescence at *both* 546 and 573 nm excitations, and since the 573 emission signal was larger than the 546 emission signal, the 573/546 ratio *increased* in response to FCCP. This was the case whether we analyzed the whole cell (Fig. 1e) or individual mitochondrion within cells. Consequently, under our conditions an increase in 573/546 ratio indicated movement of TMRM from a quenched (hydrophobic) to an unquenched (hydrophilic) compartment. Other than this difference, however, our results support and extend their results to mitochondria in living cells.

It is important to note that the spontaneous increase in TMRM fluorescence, as well as the subsequent oscillations and waves, did not occur in all neurons and never in glia cells even after several hours of very high concentration of TMRM (25 µM). Several variables seemed to enhance the likelihood of these events (e.g., higher dye concentration, longer dye incubation period, older age of cultures, higher oxygen levels of the saline), but we did not test these systematically. These variables limited our ability to establish a tighter correlation between fluorescent oscillations/waves and subsequent apoptosis.

Another variable that has been reported to be effective in inducing TMRM oscillations in cells is intense illumination [37], [38], a response that involves the induction of reactive oxygen molecules [42]. We found no evidence for this, though, in hippocampal neurons under our imaging conditions. This may have been due to our use of 2P imaging at low beam intensity, which minimized photodynamic damage to cells [28], [31]. Under these conditions, we were able to image TMRM in neurons repeatedly for extended periods without evidence of imaging-induced oscillations (cf. Fig. 2). Since oscillations were present immediately in neurons pre-incubated in dye (and kept in the dark) for 1–2 hours before imaging, this provides compelling evidence that TMRM oscillations can occur independent of illumination-induced ROS generation.

Although we found no correlation between TMRM and Ca^2+^ oscillations in hippocampal neurons, we did find that prolonged exposure to high concentrations of TMRM induced a spontaneous elevation of intracellular Ca^2+^ that preceded apoptosis (Fig. 5). Whether the Ca^2+^ response originated in mitochondria, endoplasmic reticulum (ER), or influx through the plasma membrane was not investigated here. Excessive release of Ca^2+^ from ER has been implicated in the induction of apoptosis in other cell types [43], [44] including neurons [45]. This process is initiated by cytochrome c release, from one or a few mitochondria, and binding to IP3 receptors on nearby ER inducing sustained Ca^2+^ release into the cytosol. Excessive elevation of cytosolic Ca^2+^ then triggers additional mitochondria to release cytochrome c resulting in a cascade of reactions resulting inevitably in apoptosis. The presence of proteins that bridge mitochondria and ER, called MDM complexes, are thought to facilitate this process [46].

Our most visually striking result was the slow propagating waves of TMRM fluorescence. Time-lapse movies (Movies S1, S2 and S3) revealed nuances that were difficult to convey in static images. Most remarkable was the apparent randomness of their initiation and directionality. In addition, both TMRM oscillations and waves were several orders of magnitude slower than what would be expected if they were correlated with changes in plasma membrane potential. These results add further support to the notion that the oscillations and waves were generated by intracellular events.

The average wave velocity was 0.7±0.2 µm/sec at room temperature, which is substantially slower than the diffusion coefficients of rhodamine derivatives measured in water at room temperature (D_rhodamine_ = 400–440 µm^2^/sec). Diffusion rate was relatively insensitive to changes in pH, ionic strength and dye concentration [47]. Recently, Kuimova et al. [48] estimated the cytosolic viscosity of HeLa cells to be 50 cP which is approximately 50× greater than water. Assuming that TMRM diffuses similar to rhodamine inside cells, its diffusion rate in cytoplasm would be 8–9 µm^2^/sec still substantially faster than what we measured in cells. Kuimova et al. also reported that the cytosolic viscosity increased 5-fold during photo-induced cell death. Although we found no evidence of photo-induced cell death in our experiments, it is conceivable that induction of apoptosis by TMRM increased cell viscosity, e.g., by the coagulation of proteins and nucleic acids, causing even slower diffusion of dye and contributing to generation of TMRM waves.

TMRM waves have been reported for cultured cardiac myotubes induced by either ceramide, an apoptotic agent, or micromolar elevation of intracellular Ca^2+^ [40]. Although similar to our results, there were several important differences between those experiments and ours. Each myotube wave was a solitary event (not repetitive), recorded using 50–80 nM TMRE, and correlated with an intracellular Ca^2+^ wave generated by sequential Ca^2+^ release by ER and Ca^2+^ uptake by mitochondria along the myotube. Neuronal waves, by comparison, were cyclical events (repetitive), recorded using 2.5 µM TMRM (which was the apoptosis-inducing agent), and displayed no corresponding Ca^2+^ waves. Nevertheless, it is intriguing that both types of waves correlated with induction of apoptosis and with elevation of intracellular Ca^2+^ concentration.

It is well known that high concentrations of mitochondrial dyes are capable of inducing cytotoxicity including apoptosis [49]. Our data adds to this literature by showing a correlation between TMRM oscillations and waves and subsequent apoptosis. We did not examine whether oscillations and waves could be induced by other apoptotic agents at low concentrations of TMRM, although this would be interesting to know. Likewise, we did not attempt to block TMRM-induced apoptosis with anti-apoptotic agents or treatments as only a small percentage of cells displayed oscillations or waves during the first hour or two of exposure to TMRM. Thus, one would need to extend our imaging measurements for longer periods of time to test such agents.

While the molecular events underlying TMRM oscillations and waves in neurons remain unclear, our data support the hypothesis that these phenomena resulted from movement of dye between quenched (hydrophobic) and unquenched (hydrophilic) compartments. Such movements could reflect a dissipative pattern of chemical reactions, so-called chemical waves or Belousov-Zhabotinsky reactions [50], [51], initiated by or associated with apoptosis. It is interesting to speculate further that such reactions may underlie early events in apoptosis leading to the disintegration of cytoplasmic contents.

## Supporting Information

Figure S1 **Examples of TMRM fluorescence oscillations in different neurons and calculations of the FFTs of the oscillations.** The FFT data for cells a, c and d show no dominant fluorescent oscillation frequency, whereas the FFT data for cell b shows a dominant fluorescent oscillation at 4.27 minutes.
(TIF)

Movie S1 **Time-lapse movie of TMRM fluorescence of cells in** **figure 2** **(duration: 76 min).** Notice the initial loading of mitochondria (0–40 min) followed by labeling and flickering of the processes (40–60 min) during the plateau phase and subsequent spontaneous increase in fluorescence in the cell bodies after one hour.
(MP4)

Movie S2 **Time-lapse movie of TMRM fluorescence of cells in** **figure 6** **(duration: 29 min) displaying slow propagating waves of fluorescence within neurons.**
(MP4)

Movie S3 **Time-lapse movie of TMRM fluorescence oscillations of neurons analyzed in** **figure 8** **(duration: 14 min).**
(MP4)
