# Supplementary figures and images for: Imaging of Mitochondrial and Non-Mitochondrial Responses in Cultured Rat Hippocampal Neurons Exposed to Micromolar Concentrations of TMRM
Source: PLoS One. 2013 Mar 4;8(3):e58059. doi: 10.1371/journal.pone.0058059 (PMC3587568; doi:10.1371/journal.pone.0058059)

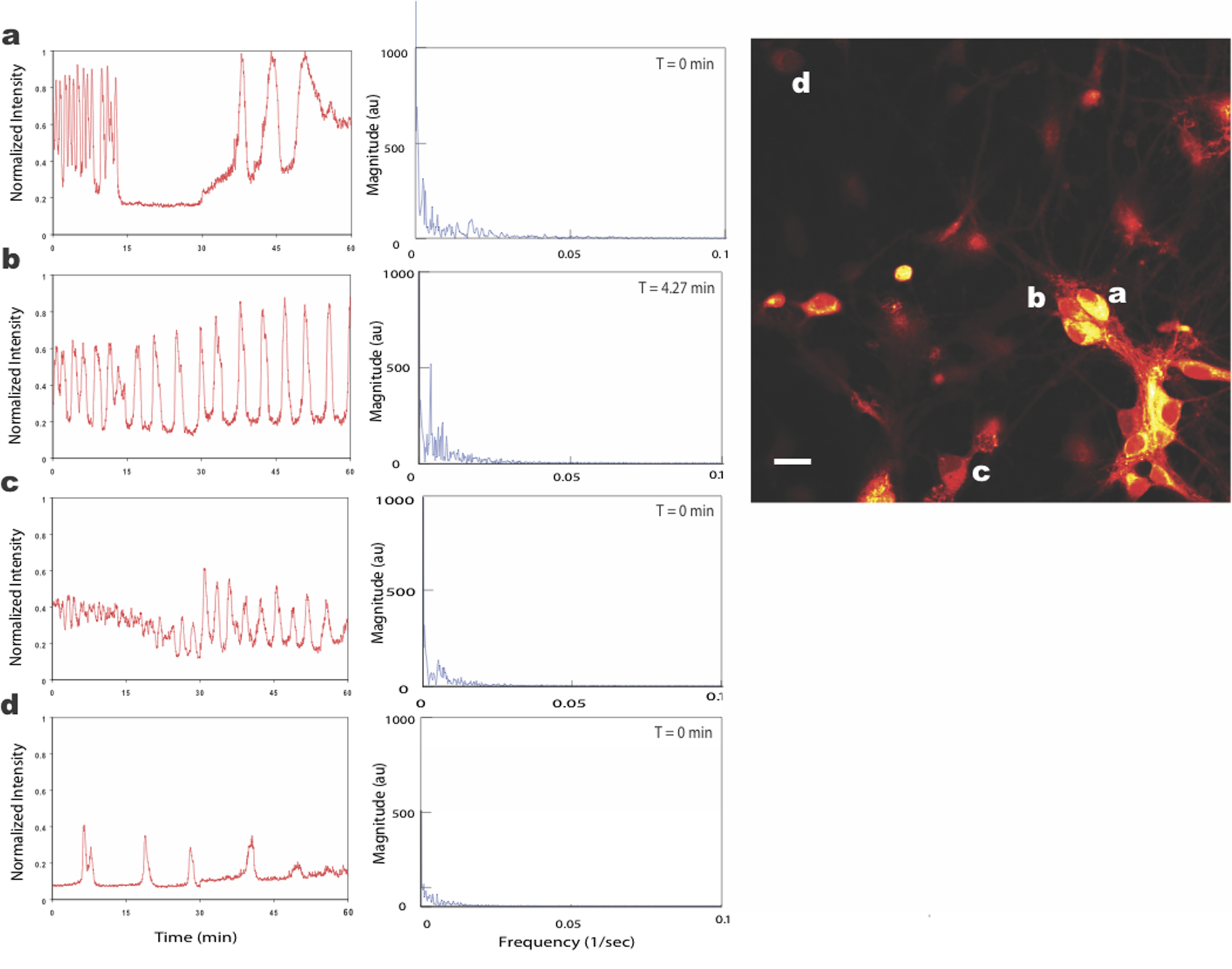

Supplement: Figure S1 — Examples of TMRM fluorescence oscillations in different neurons and calculations of the FFTs of the oscillations. The FFT data for cells a, c and d show no dominant fluorescent oscillation frequency, whereas the FFT data for cell b shows a dominant fluorescent oscillation at 4.27 minutes. (TIF) [file pone.0058059.s001.tif]
